# Supplementary material for: Validation of the NICHD Bronchopulmonary Dysplasia Outcome Estimator 2022 in a Quaternary Canadian NICU—A Single-Center Observational Study
Source: J Clin Med. 2025 Jan 22;14(3):696. doi: 10.3390/jcm14030696 (PMC11818857; doi:10.3390/jcm14030696)
Supplement: Supplementary file 1 [file jcm-14-00696-s001.zip › jcm-3384059-supplementary.pdf]

Table S1. Interventions received during NICU stay

**Interventions**	**n (%)**
Umbilical arterial catheter	73(73.7)
Umbilical venous catheter	92(92.9)
Peripherally inserted central catheter	63(63.6)
Peripheral arterial catheter	20(20.2)
Peripheral intravenous catheter	80(80.8)
Parenteral nutrition	97(98.0)
Intubation and ventilation	77(77.8)
High-frequency ventilation	46(46.5)
Antibiotics	94(94.9)
Narcotic infusion	57(57.6)
Sedatives	48(48.5)
Inhaled nitric oxide	8(8.1)
Inotropes	35(35.4)
Transfusion	63(63.6)
Postnatal steroids	23(23.2)

Table S2. Duration of interventions received during NICU stay

**Variable**	**Median (IQR)***
Ventilator support days (invasive + non-invasive)	61 (30- 87)
Intubated and ventilated days	7 (1-27)
Non-invasive ventilation days	49 (27 -67)
Oxygen days	34 (8 - 49)
Inhaled nitric oxide days	4.5 (3 - 5)
Umbilical arterial catheter days	7 (5 - 9)
Umbilical venous catheter days	7 (4-9)
Peripheral arterial catheter days	6 (4 -9)
Peripherally inserted central catheter days	22 (10 -39)
Peripheral intravenous catheter days	12 (5 -22)
Total parenteral nutrition days	15 (8- 32)
Narcotic infusion days	7 (2-16)
Sedative infusion days	5.5 (1- 17)
Antibiotic days	10.5 (4 -19)
Inotrope days	3 (2-7)

*IQR-Interquartile range

Table S3. Comparison of Receiver operating characteristic (ROC) curves across days 1, 3, 7, 14 and 28

ROC Contrast Estimation and Testing Results						
Contrast	Estimate	Standard error	95% Wald Confidence Limits		Chi-Square	P value
Day3_Grade_2/3_BPD - Day1_Grade_2/3_BPD	-0.0199	0.0238	-0.0664	0.0267	0.6975	0.4036
Day7_Grade_2/3_BPD - Day1_Grade_2/3_BPD	0.0184	0.033	-0.0464	0.0831	0.3097	0.5779
Day14_Grade_2/3_BPD - Day1_Grade_2/3_BPD	0.0404	0.0392	-0.0365	0.1174	1.0621	0.3027
Day28_Grade_2/3_BPD - Day1_Grade_2/3_BPD	0.0522	0.0407	-0.0276	0.132	1.6454	0.1996

BPD – Bronchopulmonary dysplasia
